# Dogs with Acute Myeloid Leukemia Have Clonal Rearrangements in T and B Cell Receptors

**DOI:** 10.3389/fvets.2017.00076

**Published:** 2017-05-31

**Authors:** Tracy Stokol, Gabrielle A. Nickerson, Martha Shuman, Nicole Belcher

**Affiliations:** ^1^Department of Population Medicine and Diagnostic Sciences, College of Veterinary Medicine, Cornell University, Ithaca, NY, United States; ^2^Animal Health Diagnostic Center, College of Veterinary Medicine, Cornell University, Ithaca, NY, United States

**Keywords:** acute myelogenous leukemia, canine, polymerase testing for antigen receptor rearrangements, clonality testing, phenotyping, leukemia, flow cytometry, cytochemistry

## Abstract

Clonality testing for rearrangements in the complementarity-determining region 3 of the immunoglobulin heavy chain of B lymphocytes (B cell receptor) and the T cell receptor of T lymphocytes helps distinguish between clonal and non-clonal expansions of lymphocytes. There are rare reports of clonally rearranged T and B cell receptors in dogs with acute myeloid leukemia (AML). Our objective was to determine the frequency of clonally rearranged T and B cell receptors in dogs with AML. Archived slides from historical cases of AML (from January 2010 to June 2013) and slides or liquid specimens [blood, bone marrow (BM), body cavity fluid, or tissue aspirates] from cases of AML diagnosed between June 2013 and February 2017 were used in the study. A diagnosis of AML was made on the basis of more than 20% immature neoplastic cells (“blasts”) in blood, BM, or extramedullary tissues, displaying features of myeloid differentiation. Myeloid differentiation was based on a combination of morphologic criteria, positive flow cytometric labeling for surface antigens typical of myeloid origin (e.g., CD11b, CD11c, CD14 with a general lack of expression of T or B cell markers), or positive cytochemical staining reactions for myeloid-associated enzymes (e.g., alkaline phosphatase, chloroacetate esterase). There were 63 cases of AML diagnosed during this period; however, slides or liquid specimens with sufficient DNA for testing were only obtained from 25 dogs. Affected dogs represented various breeds and were a median of 8 years old, with more male (64%) than female (36%) dogs. Common clinical signs were peripheral or internal lymphadenopathy (10/25 dogs, 40%) and hepatomegaly or splenomegaly (10/25 dogs combined, 40%). Typical hematologic findings were bi- or pancytopenia (23/25 dogs, 92%), with circulating blasts (21/25, 84%). Solitary clonal (4 B cell, 6 T cell) and biclonal (6 B and T cell) rearrangements in B or T cell receptors were found in 16 dogs (64%). Our results indicate that dogs with AML can have a high frequency of clonally rearranged T or B cell receptors, including biclonality, and clonality testing should not be used as a tool to distinguish between acute leukemia of myeloid or lymphoid origin.

## Introduction

Testing for antigen rearrangements in T and B cell receptors on genomic DNA is a useful tool to help identify clonal expansions in lymphocytes in veterinary medicine. Testing is accomplished *via* polymerase chain reactions using primers designed to amplify the complementarity-determining region 3 (CDR3) of immunoglobulin chains (usually the heavy chain or IgH) of B cells (B cell receptor) and the T cell gamma receptor (1, 2). Thus, clonality testing is known colloquially as polymerase testing for antigen receptor rearrangements (2). Amplified products have been traditionally detected using ethidium bromide and agarose gel electrophoresis (3, 4); however, higher resolution techniques, such as capillary gel electrophoresis, are supplanting this older method (1, 2, 5–7). Clonality testing is primarily used to distinguish between neoplastic and reactive lymphocyte expansions (1, 2, 6–8); however, this testing is also used as a means to phenotype lymphoid neoplasms as B or T in origin, particularly with tumors showing expression of more than one lineage with flow cytometry or immunohistochemical (IHC) staining (2, 5, 6, 8–13). The use of clonality as a phenotyping tool is being extended to myeloid neoplasms, where clonality testing has been used as a means to distinguish between acute myeloid leukemia (AML) and lymphoid neoplasms (lymphoma or leukemia) (6, 14, 15). However, we have observed clonal rearrangements in both B and T cell receptors in cases of AML in dogs and a previous study documented a clonally rearranged B cell receptor in one of three dogs with AML (3). The goal of this study was to document the frequency of clonally arranged lymphoid receptors in a cohort of dogs with AML.

## Materials and Methods

**Figure 1:**
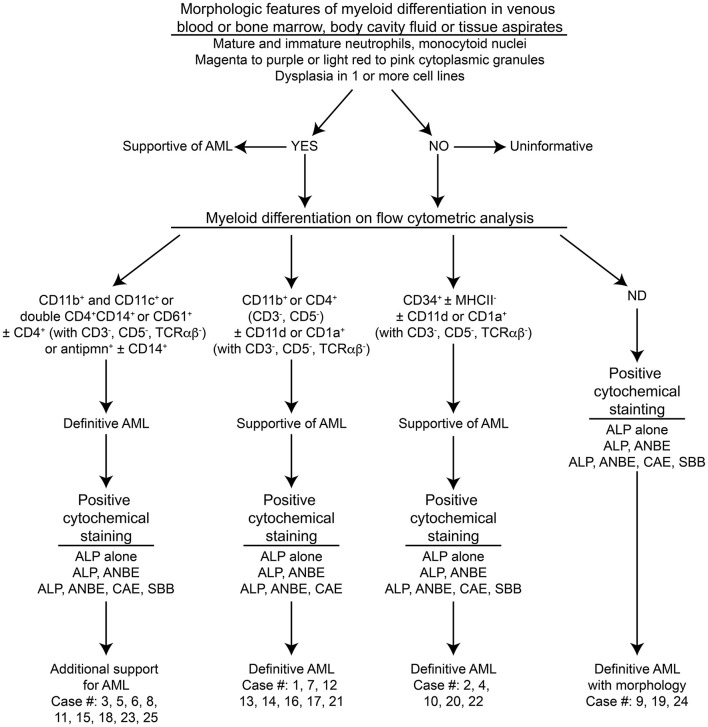
**Algorithm used to diagnose acute myeloid leukemia (AML) in the 25 dogs of this study**. This diagnostic algorithm was based on the order in which tests were generally performed in our laboratory, i.e., morphologic assessment of blood, bone marrow, or body cavity fluid or tissue aspirates, followed by flow cytometric analysis (performed routinely twice a week), followed by cytochemical staining (performed as needed). After completion of all the tests, the results were reevaluated, and a diagnosis of AML was based on the combined data. The path used to diagnose each case (#) is also shown. More details on the criteria are provided in Table 1.

This study was conceived in June 2013 and included historical cases of AML, in which archived slides were available for clonality testing (January 2010 to June 2013), and new cases of AML diagnosed from samples submitted to the Clinical Pathology laboratory in the Animal Health Diagnostic Center at Cornell University for routine diagnostic testing or for leukemia classification with phenotyping techniques, including flow cytometry and cytochemical staining (June 2013 to February 2017). A diagnosis of AML was made on the basis of greater than 20% immature neoplastic cells (“blasts”) in blood, bone marrow (BM), body cavity fluids, or extramedullary tissues, in which neoplastic cells were displaying features of myeloid differentiation. Myeloid differentiation was based on a combination of morphologic features, expression of myeloid markers on flow cytometric analysis, or expression of enzymes characteristic of myeloid origin on cytochemical staining (16) (Table 1), with the help of an algorithm centered on the order in which the diagnostic tests were usually performed (Figure 1). Whenever possible, the leukemia was further classified into subtypes, using defined World Health Organization (WHO) criteria (17, 18) (Table 2). When the subtype was difficult to determine (e.g., lack of BM results), the most likely subtype was selected and defined as “suspect.” Clonality testing was performed on archived slides from historical and newly diagnosed cases of AML or on liquid samples (blood or BM, body cavity fluid, or tissue aspirates) on newly diagnosed cases of AML.

**Table 1:** **Criteria used to support myeloid lineage of leukemia in 25 dogs**.

Test	Criteria
Morphologic features of myeloid differentiation (16, 17).	Neutrophil differentiation (immature and mature neutrophils), monocytoid nuclei, magenta to purple cytoplasmic granules that frequently overlay the nuclei, light red to pink cytoplasmic granules within a light blue cytoplasm, or dysplasia in one or more hematopoietic cell lineages (e.g., giant band neutrophils, neutrophil hypersegmentation, bizarre monocytes, megaloblastic erythroblasts, fragmented or multiple Howell-jolly bodies, giant or abnormally granulated platelets, micromegakaryocytes)
Flow cytometric markers of myeloid differentiation (16)	Neutrophilic differentiation: antineutrophil antibody, monocytic differentiation: CD14, CD11d, or CD1a (the latter two with negative T cell markers), neutrophilic or monocytic differentiation: CD11b, CD11c, or CD4 (the latter with negative T cell markers), megakaryocytes: CD61
Cytochemical stains characteristic of myeloid differentiation (16, 35)	Neutrophils: CAE, MPx, SBB, monocytes: light to strong ALP (monoblasts, differentiating monocytes), diffuse light to chunky ANBE (differentiating monocytes, monoblasts), may be positive for MPx (weaker than neutrophils) or SBB (weaker than neutrophils)

*The tests used to define criteria were run sequentially (Figure 1), but the combined results were evaluated after completion of analysis to obtain a definitive diagnosis of AML*.

*ALP, alkaline phosphatase; AML, acute myeloid leukemia; ANBE, α-naphthyl butyrate esterase; CAE, chloroacetate esterase; MPx, myeloperoxidase; SBB, Sudan Black B*.

**Table 2:** **Criteria for classification of the subtype of acute myeloid leukemia (AML) based on the World Health Organization scheme – not otherwise specified (17, 18)**.

Type of AML	Criteria
Acute myelomonocytic leukemia (M4)	≥20% cells showing neutrophilic differentiation and ≥20% showing monocytic (including promonocytes) differentiation. Neutrophil differentiation was based on one or more of the following:
	Morphologic features, i.e., mature an immature neutrophils comprised ≥20% cells in blood or bone marrow Flow cytometry: expression of neutrophil-associated markers such as antineutrophil antibody Cytochemical staining: Positive for chloroacetate esterase, myeloperoxidase, or Sudan Black B in ≥20% blasts
	Monocytic differentiation was based on one or more of the following:
	Morphologic features, i.e., mature and immature monocytes comprised ≥20% cells in blood or bone marrow Flow cytometry: expression of monocyte-associated markers, such as CD14 alone, CD4 and CD14 double positive, or CD11c or CD1a (with negative reactions for T cell markers with the latter) Cytochemical staining: Positive for alkaline phosphatase (light to strong) or diffuse light to chunky α-naphthyl butyrate esterase

Acute monoblastic or monocytic leukemia (M5)^a^	>80% monocytic lineage (monoblasts, promonocytes, monocytes) based on the above features
Mixed lineage or phenotype	Combination of morphologic features and expression of markers of more than one myeloid lineage or concurrent expression of myeloid and lymphoid lineages on flow cytometry and cytochemical staining with no clear dominant pattern

*In some dogs, the final classification was presumptive (suspect) because bone marrow was not available for the classification assays (flow cytometric analysis and cytochemical staining) or one of the classification assays was not done*.

*^a^Because both neutrophils and monocytes can express CD4 (with negative T cell markers), CD11b, or CD11c, these markers support a diagnosis of AML but alone do not differentiate between subtypes (other criteria were used instead) and are not listed here*.

### Hematologic Analysis

Hemograms were performed on all EDTA-anticoagulated venous blood (VB) samples submitted to the Clinical Pathology Laboratory at Cornell University, with exceptions noted (blood results provided by the veterinarian submitting samples). Hemogram results were obtained from an automated hematology analyzer (ADVIA 2120, Siemens Healthcare Diagnostics Inc., Tarrytown, NJ, USA), and differential leukocyte counts and blood smear examination were performed by trained medical technologists on smears stained with modified Wright’s stain, using an automated stainer (Hematek 1000, Siemens Healthcare Diagnostic Inc.). The clinical pathologist on duty reviewed the blood smears.

### Cytologic Evaluation of BM, Body Cavity Fluid, and Tissue Aspirates

Bone marrow, body cavity fluid (peritoneal or pleural), or tissue aspirates (lymph node, spleen, or liver) were performed on dogs admitted to the Cornell University Hospital for Animals and smears were prepared in the Clinical Pathology Laboratory or by the clinician obtaining the sample. For mailed-in samples, BM smears were prepared from submitted fluid samples, and these smears or submitted smears of BM, body cavity fluid, and tissue aspirates were stained with modified Wright’s stain (if not prestained) and examined by the clinical pathologist on duty. For testing done at Colorado State University (CSU), smears were submitted for review by one author (Stokol).

### Flow Cytometric Analysis of Liquid Specimens

**Figure 2:**
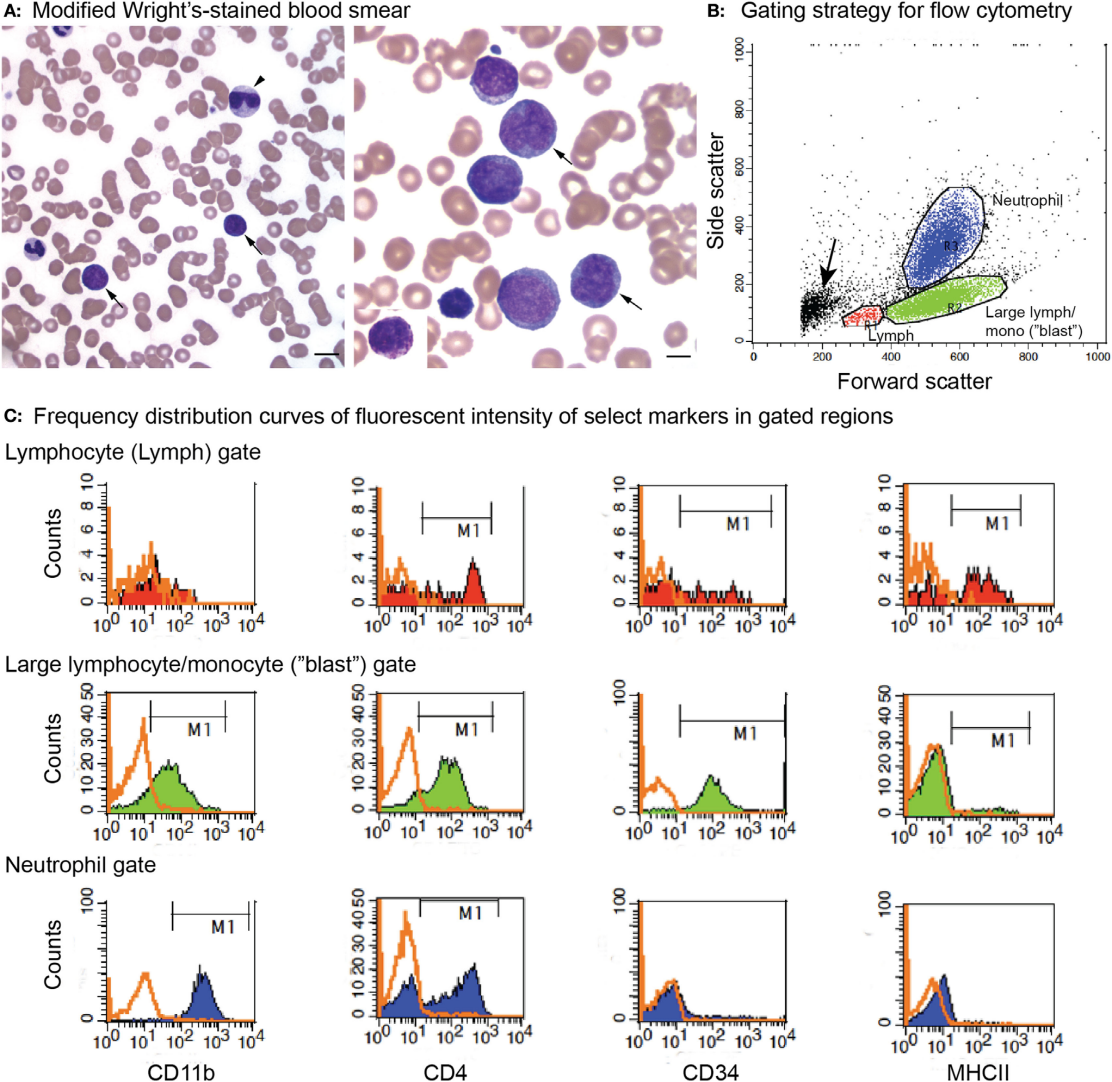
**Peripheral blood morphologic features and select flow cytometric results from a dog with acute myeloid leukemia (AML) (case 12)**. The leukemia was classified as myelomonocytic (M4) based on a combination of ≥20% mature and immature neutrophils in blood, morphologic (monocytoid nuclei), and cytochemical evidence of monocytic differentiation (positive for alkaline phosphatase or α-naphthyl butyrate esterase) in tumor cells in blood and positive expression of differentiation antigens common to neutrophils and monocytes (CD11b, CD4) on flow cytometric analysis. **(A)** Representative images of modified Wright’s-stained smears of peripheral blood. Left panel: The dog had 37% blasts (arrows) with a normal neutrophil count but a moderate left shift (2.5 × 10^6^/L immature neutrophils). Some of the immature neutrophils were dysplastic (giant band neutrophil with cytoplasmic basophilia, arrowhead). Bar = 10 µm. Right panel: Higher magnification of the blasts, with two cells displaying lobulated (monocytoid) nuclei (arrows), a morphologic feature supporting monocytic differentiation. Bar = 5 µm. Right panel inset: Rare blasts had moderate numbers of purple cytoplasmic granules. We have previously reported on the presence of these “granular blasts” in dogs with AML (16). **(B)** Flow cytometric gating strategy for analysis of marker expression of the tumor cells in dogs with AML. Cells were gated as small lymphocytes (red, lymph), large lymphocytes/monocytes (green, large lymph/mono), or neutrophils (blue) based on forward and side scatter characteristics. In this case, blasts fell mostly in the large lymphocyte/monocyte (“blast”) gate. With some leukemias, only one or two (“blast” with lymphocyte or neutrophil) gates could be generated. The small events (arrow) were likely dead cells or fragments of dead cells. **(C)** Events within each gate were displayed on a frequency distribution curve (histogram plot) of counts versus fluorescent intensity of the antibody against the marker of interest. Cells positive for the marker were identified by the M1 marker region, corresponding to the location of the solid curve relative to the isotype control (overlaid orange open curve). Cells within the large lymphocyte/monocyte or “blast” gate (middle row) were positive for the neutrophil or monocyte marker, CD11b (far left column), and the neutrophil or activated monocyte marker, CD4 (middle left column). They were also positive for the stem cell marker CD34 (middle right column) but negative for MHCII (far right column). A few small tumor cells were present in the small lymphocyte gate (top row, CD34-positive cells); however, most of the cells in this gate were positive for T (CD3 and CD5) or B (CD21 and CD22) cell markers (not shown) and MCHII, as expected for normal lymphocytes. The CD4 expression is likely mostly on T lymphocytes. Cells within the neutrophil gate (lower row) had staining characteristic of neutrophils, i.e., positive for CD11b and CD4 and negative for CD34 and MCHII, serving as an internal positive and negative control for these antigens. Note, the intensity of CD11b and CD4 (distance along the X-axis) was lower for the blasts than the neutrophils, which can be a helpful feature to identify subpopulations of blasts among residual normal cells.

Flow cytometric analysis was performed on VB or BM or tissue aspirates at Cornell University in most dogs as previously described in detail (16), using conjugated and unconjugated antibodies, with the addition of antibodies against CD18 (a pan-leukocyte marker), CD61 (a platelet or megakaryocyte marker), and mature neutrophils (Table 3). In brief, cell populations were identified and gated on their forward and side scatter characteristics, with tumor cells (blasts) typically falling into the large lymphocyte or monocyte gate (Figure 2). In several cases, tumor cell events spanned the small lymphocyte and large lymphocyte/monocyte regions. Cells within the gated region of interest (tumor cell gate) were considered positive for a marker if greater than or equal to 20% of the neoplastic cells were labeled with the antibody (16), with the exception of CD34, in which normal leukocytes are negative for this marker and >5% of labeled cells was considered a positive reaction. Not all markers were applied to samples from each dog, and provided negative reactions in this study are mostly confined to the pan-leukocyte antigen CD18, MHCII [as a marker of AML (16)], and T or B cell markers (as a means to exclude lymphoid origin). Flow cytometric analysis was done on one case at CSU as part of their phenotyping diagnostic service. The service typically tests for CD45, CD18, CD3, CD5, CD4, CD8, CD21, CD14, MHCII, and CD34 using conjugated antibodies.

**Table 3:** **Antibodies used in flow cytometry at Cornell University to label antigens on tumor cells in liquid samples (blood or bone marrow, body cavity fluid, or tissue aspirates) from dogs with acute myeloid leukemia**.

Antigen	Labeled cells	Clone	Conjugate	Source^a^
CD45	Pan-leukocyte	YKIX716.13	PE	AbD Serotec
CD18	Pan-leukocyte	CA1.4E9	AF647	AbD Serotec
CD3	T cells	CA17.2A12	FITC	AbD Serotec
CD5	T cells	YKIX322.2	PE	AbD Serotec
CD4	T helper/regulatory cells, neutrophils, activated monocytes	YKIX302.9	FITC	AbD Serotec
CD8α	Cytotoxic T cell	YCATE55.9	PE	AbD Serotec
CD28	T cells	B58	APC	eBioscience
TCRαβ	T cells	CA15.8G7	None	UC-Davis
CD21	B cells	B-ly4	PE	BD-Biosciences
CD22	B cells	RFB4	PE	Abcam
CD94	Natural killer cell, cytotoxic T cell	HP-3D9	APC	eBioscience
CD14	Monocytes	Tük4	PE	Dako
CD34	Stem cell	1H6	PE	BD-Biosciences
MHCII	Lymphocytes, monocytes	YKIX334.2	FITC	AbD Serotec
CD80	Monocytes, neutrophils	16-10A1	APC	eBioscience
CD11b	Neutrophils, monocytes	CA16.3E10	None	AbD Serotec
CD11c	Monocytes, neutrophils,	CA11.6A1	None	AbD Serotec
CD11d	T subset, some monocytes	CA11.8H2	None	AbD Serotec
CD1a	T subset, B subset, monocytes	CA13.9H11	None	UC-Davis
Anti-pmn	Neutrophil	CAD048A	None	VMRD
CD90 (Thy-1)	Lymphocytes, monocytes, stem cells, eosinophils	CA1.4G8	None	UC-Davis
CD61	Platelets	SZ21	PE	Beckman-Coulter

*AF, alexafluor; PC, allophycocyanin; FITC, fluorescein isothiocyanate; PE, phycoerythrin; pmn, neutrophil*.

*^a^Abcam, Cambridge, MA, USA; AbD Serotec, now part of Bio-Rad, Hercules, CA, USA; BD-Biosciences, Franklin Lakes, NJ, USA; Beckman-Coulter, Fullerton, CA, USA; Dako, now part of Agilent Technologies, Santa Clara, CA, USA; UC-Davis: Peter Moore, University of California-Davis, Leukocyte Antigen Biology Laboratory, Davis, CA, USA; VMRD, Pullman, WA, USA*.

### Cytochemical Staining of Smears of Blood or BM, Body Cavity Fluid, or Tissue Aspirates

Cytochemical staining was performed at Cornell University, using various combinations of alkaline phosphatase (ALP), α-naphthyl butyrate esterase (ANBE), chloroacetate esterase (CAE), myeloperoxidase, and Sudan Black B, as previously described (16). Not all cytochemical stains were done on every case. As for our previous study on acute leukemia, 100 neoplastic cells were counted when possible, and tumor cells were considered positive if greater than or equal to 3% were positive for the cytochemical stain (16).

### Clonality Testing

**Figure 3:**
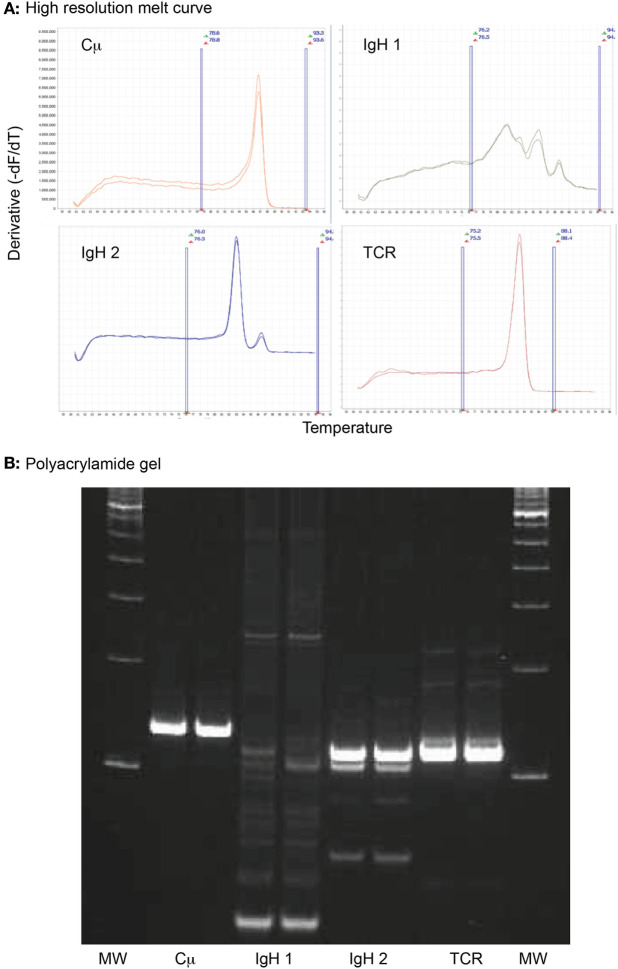
**High-resolution melt curve analysis of amplified DNA products retrieved from an archived venous blood smear of a dog with acute myeloid leukemia (case 1)**. **(A)** Sharp peaks in the negative derivative curves yielded from the normalized DNA melt curve showed that the tumor cells had clonal rearrangements in complementarity-determining region 3 (CDR3) of the immunoglobulin heavy chain with primer set 2 (IgH 2) and the CDR3 of T cell gamma receptor (TCR), i.e., a biclonal rearrangement. The multiple broad peaks with the first primer set for the CDR3 of the immunoglobulin heavy chain (IgH 1) is compatible with a polyclonal product. The positive control (Cμ) yielded the expected single peak. All reactions were run in duplicate. Note that all curves have the same Y- and X-axis. The area between the vertical blue lines represent the active melt curve region, in which the calculations are performed. **(B)** A 10% polyacrylamide gel was performed on the sample to verify the results of the high-resolution melt curve analysis. This showed a broad smear (polyclonal) with primer set 1 for the heavy chain CDR3 (IgH 1), a strong clonal band with primer set 2 for the heavy chain CDR3 (IgH 2), and a strong single clonal band with the T cell gamma receptor CDR3 primer set (TCR). The positive control (Cμ) yielded a single strong product, as expected. All reactions were run in duplicate. MW represents the molecular weight marker (100 base pair ladder).

Clonality testing was done by the Molecular Diagnostics Laboratory in the Animal Health Diagnostic Center at Cornell University or the Clinical Immunology Laboratory at CSU (2). In the Molecular Diagnostics Laboratory, we used high-resolution melt curve analysis, followed by polyacrylamide gel electrophoresis for verification as needed. In brief, genomic DNA was retrieved from cytologic or blood smears by scraping material with a scalpel blade into a 1.5-mL polypropylene snap cap tube. DNA was extracted from the retrieved material or liquid samples using DNeasy DNA extraction columns (Qiagen, Germantown, MD, USA). The DNA was amplified using MeltDoctor™ HRM Master Mix, according to the manufacturer’s recommendations (Life Technologies, Carlsbad, CA, USA). The mixture includes a fluorescent dye that intercalates with double-stranded DNA. Four primer sets (final primer concentration of 0.52 µM) were used, including two sets to amplify the IgH CDR3 region, one set to amplify the T cell gamma receptor CDR3 region, and one set to amplify the constant region of the IgM heavy chain (Cμ) as an internal positive control. Primer sequences were based on previously published data (3). All reactions were run in duplicate. Cycling conditions were 95°C for 15 min, followed by 40 cycles at 94°C for 8 s, 60°C for 10 s, and 72°C for 15 s. As the temperature increases with each cycle, the DNA melts yielding single-stranded products, leading to the release of the intercalated fluorescent dye and a decrease in fluorescence. The melting temperature is predictable and based on the sequence of nucleotides in the starting DNA. The rate of decrease in fluorescence is monitored in real time, producing a melt curve, where normalized fluorescent intensity is plotted against temperature. This is converted to a negative derivative curve, which displays fluorescent intensity versus the temperature at which 50% of the DNA has melted (19). Clonal populations result in a single- or double-sharp peak on the derivative curve, whereas polyclonal populations produce multiple, broader, or wider derivative curves due to the different DNA sequences melting at different temperatures (Figure 3). Melt curves were generated using the default setting of the software (High Resolution Melt Software v3.0.1, Applied Biosystems, Foster City, CA, USA). When needed, reaction products were resolved with a 10% polyacrylamide gel in a Tris buffer (89 mM Tris, 89 mM boric acid, 2 mM EDTA, pH 8.4), and bands were visualized by immersion in a DNA dye (3× GelRed Nucleic Acid Gel Stain, Biotium, Fremont, CA, USA). Bands were visualized by UV transillumination and photographed (Figure 3).

Internal validation of the procedure was performed on samples from 28 cases, including 18 cases of lymphoid neoplasia (12 B cell, 6 T cell) and 10 reactive conditions. Clonality was identified in 14 of 18 (10/12 B cell and 4/6 T cell) samples with lymphoid neoplasia, yielding a sensitivity of 78%. Non-clonal results were seen in 9 of 10 reactive conditions, yielding a specificity of 90%. The one reactive sample with B cell clonality was diagnosed with lymphoid hyperplasia on a splenic aspirate. The positive result was confirmed with repeat testing at CSU. The sensitivity of our assay falls within that reported for similar primer sets in other studies (5–7, 10, 20).

### Ethics Statement

Residual samples were used (archived slides or that remaining after routine clinical pathologic testing); therefore, approval from the Institutional Biosafety and Animal Use Committee or client consent was not required.

## Results

Over the time period of this study (June 2010 to February 2017), we diagnosed AML in 63 dogs; however, archived or fresh specimens were only available for 27 dogs. The yield of DNA from samples of 2 dogs was too low to provide results for clonality testing, and these dogs were excluded, leaving a total of 25 dogs from our institution (#1–25) for inclusion in the study. Dr. A. Avery (CSU, with consent from the referring veterinarian, Dr. C. Herrera) kindly provided historical information and flow cytometric and clonality results from case #25. Dogs were of various breeds, consisting mostly of Labrador Retrievers (*n* = 5) with fewer German Shepherd dogs (*n* = 3), Golden Retrievers (*n* = 2), Boxers (*n* = 2), and Rottweilers (*n* = 2). Single dogs represented other pure breeds and mixed breeds. Dogs were generally older, with a median age of 8 years (range, 1.9–12 years). There were 16 male dogs (64%), most of which were neutered, and 9 female dogs (36%), most of which were spayed. Presenting clinical signs were vague and not specific in many dogs, but some consistent findings were mild to moderate lymphadenopathy, affecting single or multiple peripheral or internal lymph nodes (*n* = 10), organomegaly (splenomegaly, hepatomegaly, or both, *n* = 10), suspect leukemia (e.g., leukocytosis, big buffy coat, *n* = 9), and previously documented single or multiple cytopenias (*n* = 5). Two dogs had a prior diagnosis of lymphoid neoplasia (lymphoma and lymphoblastic leukemia) (Table 4).

**Table 4:** **Signalment and presenting clinical signs in 25 dogs with acute myeloid leukemia in which clonality testing was performed**.

Dog	Breed	Age (years)	Sex	Clinical signs
1	Springer Spaniel	9.7	MN	Vomiting, straining to defecate, dyspnea, fever
2	German Shepherd dog	6.5	MN	Weight loss (10 kg), inappetence, diarrhea, hepatosplenomegaly
3	German Shepherd dog	10	FS	Polydipsia, inappetence, fever, firm lymph nodes, splenomegaly
4	Boxer	8	FS	Neurologic disease
5	Collie	5	FS	Anemia, hepatosplenomegaly
6	Golden Retriever	8	MN	Anorexia, vomiting, moderate peripheral lymphadenopathy
7	Mixed breed	11	MN	Inappetence, splenomegaly, leukocytosis
8	Great Dane	7.5	MN	Moderate to marked peripheral lymphadenopathy (mandibular, prescapular), diagnosed as non-B non-T lymphoma on prior lymph node aspirate and biopsy
9	Boxer	3	FS	Fever, splenomegaly
10	Labrador Retriever	3.5	FS	Prior diagnosis of “lymphoblastic leukemia,” splenomegaly
11	Labrador cross	8	MN	Dyspnea, “lymphocytosis,” mild peripheral lymphadenopathy
12	German Shepherd dog	9	MN	Anorexia, lethargy, marked “monocytosis”
13	Golden Retriever	11	MN	Routine seizure check, neoplastic cells noted in circulation
14	Bernese Mountain dog	2	MN	Thrombocytopenia evaluation
15	Labrador Retriever	10	MN	Vomiting, diarrhea, “lymphocytosis,” mild to moderate peripheral lymphadenopathy
16	Rottweiler	9	FS	Vomiting, inappetence, moderate peripheral and thoracic lymphadenopathy, hepatosplenomegaly
17	Cavalier King Charles Spaniel	6	FS	Leukocytosis, pleural effusion, mild peripheral lymphadenopathy
18	Labrador Retriever	8	MN	Inappetence, straining to defecate and liquid diarrhea, one vomiting episode, marked leukocytosis
19	Labrador Retriever	4	MN	Acute collapse, pancytopenia, hepatosplenomegaly
20	West Highland White Terrier	3	MN	Anorexia, lethargy, pancytopenia
21	Border Collie mix	10	MN	Mandibular lymphadenopathy, mild abdominal lymphadenopathy and splenomegaly
22	Rottweiler	1.9	F	Mandibular and popliteal lymphadenopathy, moderate hepatosplenomegaly
23	Labrador Retriever	2	M	Anemia, prescapular and popliteal lymphadenopathy, ascites
24	Mixed breed	12	FS	Seizures, hypoglycemia, large buffy coat
25	Labradoodle	9	MN	Moderate popliteal lymphadenopathy

*F, intact female; FS, female spayed; M, intact male; MN, male neutered*.

*Underlined dog numbers were included in our previous publication on ALP staining in acute myeloid leukemia (16). Case 2 was also presented in the February 2015 American Society of Veterinary Clinical Pathology on-line rounds (available to members only at https://www.asvcp.org/)*.

Hemogram results were available for all dogs, two of which were provided by the referring veterinarian (with no concurrent blood smear) (Table 5). Most of the dogs were anemic (21/25, 84%), which was normocytic and normochromic in 57% (12/21) and non-regenerative (absolute reticulocyte counts within reference intervals) in 79% of anemic dogs in which a reticulocyte count was performed (15/19, including one dog with a marginally increased reticulocyte count of 93 × 10^6^/L). Four dogs had a mildly to moderately regenerative anemia. Red blood cells were macrocytic and normochromic or hypochromic in eight dogs. In two of these dogs, the blood samples were mailed to the laboratory, and the macrocytic hypochromic red blood cell indices could be an artifact of red blood cell swelling with storage. Two dogs had evidence of erythroid dysplasia, which could be contributing to the macrocytosis. A normoblastosis was detected in six dogs. Leukocytosis was more common (14/25, 56%) than leukopenia (8/25, 32%) and, in most dogs, the leukocytosis was due to high numbers of blasts (12/14, 86%). Blasts were seen in 22 dogs overall (88%), with rare circulating blasts identified on blood smear examination, but not included in the differential cell count, in 4 dogs. A neutrophilia was seen in three dogs (12%), all of which had an increase in immature neutrophils (left shift), with a monocytosis in seven dogs (28%) and lymphocytosis in three dogs (12%). Ten dogs were neutropenic (40%), only one of which had a left shift, and thrombocytopenia was seen in most dogs (22/25, 88%). The mean platelet volume was high in 14 of 18 dogs (78%) in which a result was obtained. Seven dogs were pancytopenic (28%), with pancytopenia defined as a non-regenerative anemia, neutropenia, and thrombocytopenia (Table 5). Of the 23 dogs in which blood smears were reviewed, 7 dogs (30%) had cytologic evidence of dysplasia in one or more cell lineages in blood (Table 6).

**Table 5:** **Hematologic findings in 25 dogs with acute myeloid leukemia in which clonality testing was performed**.

Dog	HCT, L/L	Hg, g/L	MCV, fL	MCHC, g/L	Retic, %	Abs Retic, x10^6^/L	nRBC/100 WBC	WBC, ×10^6^/L	PMN, ×10^6^/L	BAND PMN, ×10^6^/L	LYMPH, ×10^6^/L	MONO, ×10^6^/L	BLAST, ×10^6^/L	PLAT, ×10^6^/L	MPV, fL
1	**0.22**	**72**	74	330	0.5	16	0	**279.3**	**0**	0	**0**	**0**	**276.5**	**<30** ^a^	NA
2^b^	**0.11**	**37**	76	340	ND	ND	0	**4.1**	3.3	0	**0.7**	0.1	0	**170** ^a^	ND
3	**0.25**	**63**	71	330	0.5	16	1	9.0	**0.2**	0.1	1.3	**0**	**7.0**	**44**	**22**
4	**0.36**	**113**	**79**	**310**	**2.3**	**107**	**4**	**32.8**	4.9	**2**	1.6	0.3	**24**	**82**	**23.9**
5	**0.11**	**26**	71	**250**	**4.4**	65	**4**	**85.6**	**43.6**	**2.6**	3.4	**28.2**	**7.7**	252	**19.5**
6	**0.28**	**96**	70	340	**2.1**	82	0	**102.6**	**2.1**	**3.1**	4.1	**25.7**	**67.7**	>250^a^	ND
7	**0.30**	**100**	74	330	**4.3**	**173**	**15**	**285.7**	5.7	**2.9**	**0**	**0**	**274.3**	**70**	**20.6**
8	0.52	173	68	340	ND	ND	0	6.0	3.9	0	1.3	0.2	0	197	9.6
9	0.45	162	66	360	ND	ND	0	**21.6**	**1.9**	0	1.9	**21.0**	**2.8**	**Low**	NA
10	**0.21**	**69**	**77**	330	**5.3**	**145**	0	**151.0**	**18.1**	**15.1**	1.5	**1.5**	**114.8**	**52**	**17.4**
11	**0.23**	**81**	72	350	**2.9**	**93**	**4**	**4.2**	3.6	0	**0.3**	0.2	**0.1**	**153**	**14.4**
12	**0.26**	**86**	**77**	**320**	0.4	13	**4**	**25.4**	**10.7**	**2.5**	2.3	0.5	**9.4**	**16**	**19.1**
13	**0.38**	**117**	**81**	**310**	0.9	42	0	**3.7**	**1.5**	0	2.2	**0**	0	**169**	13.6
14	**0.36**	**125**	76	350	0.5	24	0	**3.5**	2.8	0	**0.5**	**0**	0	**54**	13.2
15	**0.22**	**70**	**78**	**320**	**2.1**	60	0	**47.6**	**1.4**	0	**0**	**2.9**	**43.3**	**48**	**18.0**
16	0.45	151	74	330	ND	ND	1	**167.5**	3.4	**5.0**	1.7	**0**	**155.7**	**57**	**18.6**
17	**0.40**	**131**	**77**	330	0.4	19	0	**227.6**	4.6	**2.3**	**9.1**	**0**	**211.7**	**108**	**17.5**
18	**0.25**	**85**	70	340	1.0	34	0	**108.2**	**1.1**	0	**6.5**	**2.2**	**98.5**	**79**	**18.2**
19	**0.27**	**89**	73	330	**1.6**	41	0	10.0	6.8	0	2.3	0.6	Rare	**<30** ^a^	NA
20	**0.19**	**63**	**80**	**260**	0.9	23	0	**0.7**	**0.1**	0	**0.6**	**0**	Rare	**9**	**18.7**
21	0.41	141	72	330	ND	ND	0	**3.5**	**2.0**	0	1.2	0.2	Rare	**109**	**16**
22	**0.30**	**99**	68	340	**0**	**2**	0	13.5	6.2	**3.0**	1.4	0.8	**0.9**	**32**	**16**
23	**0.11**	**38**	74	340	ND	ND	**2**	**36.1**	5.1	**0.7**	3.6	**5.4**	**21.3**	**12**	13.4
24	**0.34**	**84**	**100**	**250**	**3.9**	**132**	0	**202**	8.1	**2.0**	**6.1**	**22.2**	**163.6**	**<30^a^**	NA
25^b^	**0.37**	**56**	66	350	1.1	64	0	**4.6**	**2.3**	0	1.9	0.6	Rare	**142**	ND
RI	0.41–0.58	141–201	64–76	330–360	0.2–1.5	11–92	0–1	5.7–14.2	2.7–9.4	0–0.1	0.9–4.7	0.1–1.3	0	186–545	8.4–14.1

*HCT, hematocrit; Hg, hemoglobin; MCV, mean corpuscular volume; MCHC, mean corpuscular hemoglobin concentration; Retic, reticulocyte count; Abs Retic, absolute reticulocyte count; nRBC, nucleated red blood cells; WBC, leukocyte count (corrected if more than 5 nRBC/100 WBC); PMN, segmented neutrophil count; BAND PMN, immature neutrophil count, including band neutrophils and earlier forms; LYMPH, lymphocyte count; MONO, monocyte count; BLAST, Blast count; PLAT, platelet count; MPV, mean platelet volume; ND, not done; NA, not available; RI, reference interval; AML, acute myeloid leukemia; ALP, alkaline phosphatase*.

*Bolded items indicate values above or below the reference intervals established at Cornell University for the ADVIA 2120 hematology analyzer*.

*Underlined dog numbers were included in our previous publication on ALP staining in AML (16). Case 2 was also presented in the February 2015 American Society of Veterinary Clinical Pathology on-line rounds (available to members only at https://www.asvcp.org/)*.

*^a^Estimated from a smear. The automated count was unreliable due to platelet clumping or cytoplasmic fragments from tumor cells interfering with count. When an estimated platelet count was used or hemogram results were provided from the referring veterinarian, the MPV was ND or not provided with the hemogram results (NA)*.

*^b^Hemogram results provided by veterinarian, with no accompanying blood smear for review. For #25, the hemogram results were from the first presentation at the oncologist and accompanied the flow cytometric results on the lymph node aspirate performed at Colorado State University (Table 6). Subsequent hemograms from this dog (provided by the oncologist) showed progressive disease, with increased blasts, despite treatment for AML. The dog was euthanized 2.5 months after diagnosis, and select results from the hemogram performed at Cornell University upon euthanasia are provided in Table 6*.

**Table 6:** **Morphologic findings from blood or cytologic smears and results from flow cytometric labeling, cytochemical staining, and clonality testing in 25 dogs with AML**.

Dog	Criteria					AML Subtype^b^	Clonality
	Defining features on venous blood (VB), bone marrow (BM), cavity fluid or tissues		Flow cytometric results		Cytochemical reactions^a^		
	VB	BM, body cavity fluid or tissues	Positive	Negative			
1	None	ND	VB: CD45, CD18, CD34 (66%), CD11b, CD11d, CD1a, CD90	VB: MHCII, CD3, CD5, TCRαβ, CD21, CD22	VB: ALP (18% light), ANBE (18%), CAE (4%)	M5	VB: B (set 2) and T clonal (Figure 2)

2	ND (only report provided)	BM: 40–45% myeloid blasts, trilineage dysplasia	BM: CD45, CD34 (55%), CD11d, CD1a, CD90	BM: MHCII, CD18, CD3, CD5, TCRαβ, CD21, CD22	BM: ALP (64% strong)	Mixed lineage	BM slide: T clonal
					Spleen: negative		
		Spleen: >80% blasts (suspect erythroid)					

3	Monocytoid nuclei, purple granules	ND	VB: CD45, CD34 (54%), CD14, CD11b, CD11c, CD11d, CD1a, CD90	VB: MHCII, CD3, CD5, TCRαβ, CD21, CD22	VB: ALP (light), ANBE (8%)	M5	VB slide: T clonal

4	None	ND	VB: CD45, CD34 (82%), CD5 (25%), CD90	VB: CD3, TCRαβ, CD21	VB: ALP (strong)	Suspect M5	VB slide: non-clonal

5	Trilineage dysplasia	ND	VB: CD45, CD34 (17%), CD11b, CD11c	VB: CD3, CD5, TCRαβ, CD21	ND	M5	VB slide: B clonal (set 2)

6	Monocytoid nuclei, light red granules, dysplasia (mono)	ND	VB: CD45, CD34 (79%), CD4, CD5 (28%), CD14, CD11b, CD11c, CD11d, CD90	VB: MHCII, CD3, TCRαβ, CD21, CD22	VB: ALP (strong), CAE (7%)	M5	LN slide: T clonal (also CSU)
		LN: >80% blasts					

7	Variable blasts (some monocytoid, others erythroid)	BM: 98% blasts, some with purple granules	BM: CD45, CD34 (79%), CD5 (26%), CD11b, CD1a, CD90	BM: CD3, TCRαβ, CD21	BM: ALP (strong)	M5	BM slide: B (set 2) and T clonal

8	None	BM: 22–30% blasts	BM: CD45, CD18, CD34 (13%), CD11b, CD11c, CD90	BM: MHCII, CD3, TCRαβ, CD21, CD22	BM: ALP (>90% strong), ANBE (>90%)	M5	LN slide: non-clonal
		LN: >90% blasts	LN: CD45, CD18, CD34 (64%), CD90	LN: MHCII, CD3, TCRαβ, CD21, CD22	LN: ALP (49% moderate), ANBE (39%)		

9	Monocytoid nuclei	BM: 57% blasts, monocytoid nuclei	ND	ND	BM: ALP (strong)	Suspect M5	BM slide: non-clonal

10	Monocytoid nuclei, dysplasia (pmn, mono)	BM: 90% blasts, dysplasia (erythroid, pmn)	VB and BM: CD45, CD34 (29%)	VB and BM: MHCII, CD3, CD5, CD21, CD22	BM: ALP (strong), ANBE, CAE (58%), SBB (6%)	M4	BM slide: B clonal (set 2)

11	None	BM: 35% blasts	BM: CD45, CD18, CD11b, CD11c	BM: MHCII, CD34 (5%), CD3, CD5, TCRαβ, CD21, CD22	BM: ALP (60% strong), ANBE (18%), CAE (18%)	Suspect M4	BM slide: T clonal, B inconclusive

12	Trilineage dysplasia	ND	VB: CD45, CD18, CD34 (98%), CD4, CD11b, CD90	VB: MHCII, CD3, CD5, TCRαβ, CD21, CD22	VB: ALP (100% strong), ANBE (100%)	M4	VB slide: B clonal (set 2)

13	None	BM: 99% blasts	BM: CD45, CD18, CD34 (97%), CD11b, CD11d, CD1a, CD90	BM: MHCII, CD3, CD5, TCRαβ, CD21, CD22	BM: ALP (100% strong), ANBE (4%)	M5	BM slide: B (set 1) and T clonal
		LN: >90% blasts					

14	None	BM: 83% blasts with pink granules	BM: CD45, CD18, CD34 (18%), CD11b	BM: MHCII, CD3, CD5, TCRαβ, CD21, CD22	BM: ALP (3% moderate), ANBE (29%), CAE (12%)	M5	BM slide: non-clonal

15	Red to purple granules	BM: >90% blasts	VB: CD45, CD34 (36%), CD18, CD14, CD11b, CD11c, CD1a, CD90	VB: MHCII, CD3, CD5, TCRαβ, CD21, CD22	VB: ALP (54% moderate), ANBE (24%) BM: ALP (100% strong), ANBE (34%), CAE (12%)	M5	VB slide: B (set 2) and T clonal

16	Magenta to purple granules	BM: >90% blasts	BM: CD45, CD18, CD34 (95%), CD22 (71%), CD11b, CD11d, CD90	BM: MHCII, CD3, CD5, TCRαβ, CD21	BM: ALP (100% strong), ANBE (99%), CAE (21%)	M4	VB slide: T clonal

17	None	BM: ND PLF: >80% blasts	VB: CD45, CD34 (37%), CD5 (34%), CD11b, CD90	VB: MHCII, CD3, TCRαβ, CD21, CD22	VB: ALP (100% moderate), ANBE (54%), CAE (4%) PLF: ALP (100% moderate), ANBE (7%), CAE (7%)	M5	VB and PLF slides: non-clonal

18	None	ND	VB: CD45, CD18, CD34 (91%), CD4, CD11b, CD11c, CD11d, CD90	VB: MCHII, CD3, CD5, TCRαβ, CD21, CD22	VB: ALP (11% moderate)	M5	VB slide: B (set 2) and T clonal

19	None	BM: 99% blasts	ND	ND	VB: ALP (100% strong), ANBE (33%), CAE (4%)	M5	BM slide: non-clonal

20	None	BM: 32–70% blasts	BM: CD45, CD18, CD34 (94%), CD3 (35%), CD22 (35%)	BM: MHCII, CD5, TCRαβ, CD21	BM: ANBE (>80%) CAE (59%)	Mixed lineage	BM slide: B clonal (set 2)

21^c^	None	BM: ND	LN: CD45, CD34 (36%), CD4	LN: MHCII, CD3, CD5, CD21, CD22	LN: ALP (75% strong), ANBE (4%)	M5	LN slide: B clonal (set 1)
		LN: >80% blasts, magenta granules					

22^c^	Dysplasia (pmn, eos, platelets)	BM: ND	VB: CD45, CD18, CD34 (39%) and MHCII double positive, CD1a	VB: CD3, CD5, TCRαβ, CD21, CD22	VB: ALP (>80%), ANBE (33%), CAE (33%)	M4	LN slide: non-clonal
		LN: 27% blasts, dysplasia (pmn, eos)					

23	Trilineage dysplasia	BM: ND	VB: CD45, CD18, CD34 (6%), CD3 (62%), CD61 (60%)	VB: MHCII, CD5, TCRαβ, CD21, CD22	VB: ALP (100% moderate), ANBE (9%)	Mixed lineage	PTF fluid: T clonal
		PTF: >20% blasts					

24	None	ND	ND	ND	VB: ALP (98% moderate), ANBE (24%), CAE (24%)	M4	VB slide: non-clonal

25	Monocytoid nuclei, dysplasia (mono)	BM: 95% blasts	VB: CD45, CD18, CD34 (41%), CD4, CD14 (26% double positive with CD4), CD11c, CD11d, CD1a, CD90	VB: MHCII, CD3, CD5, TCRαβ, CD21, CD33	VB: ALP (63% light), ANBE (90%), CAE (35%)	M4	LN slide: B and T clonal (CSU)
		LN: >20% blasts, monocytoid nuclei, dysplasia (mono)	BM: ND	BM: ND	BM: ALP (85% moderate), ANBE (68%), CAE (44%)		
			LN: CD34 (6%), CD14 and CD4 double (CSU)	LN: MHCII, CD3, CD5, CD21 (CSU)	LN: ALP (65% moderate), CAE (17%)		

*All results were obtained from testing done at Cornell University unless stated otherwise in parentheses. Note that not all of the antibodies provided in Table 3 were used in every dog, and negative results for flow cytometric analysis was confined to T (CD3, CD5, TCRαβ), B (CD21, CD22), or stem (CD34) cell markers, the pan-leukocyte marker (CD18), and MHCII. If results for the latter markers are not provided, then the antibody against the marker was not tested in that case. If a marker or cytochemical reaction is not listed, it means the test result was negative (usual scenario) or not performed. When available, the percentage of blasts with positive cytochemical reactions (and the strength of the reaction for ALP) is provided*.

*NA, not available for review with no report provided; ND, not done; PLF, pleural fluid; PTF, peritoneal fluid; M4, acute myeloid leukemia – not otherwise specified, myelomonocytic; M5, acute myeloid leukemia – not otherwise specified, monocytic or monoblastic; CSU, Colorado State University; pmn, neutrophil; mono, monocyte; eos, eosinophil; IHC, immunohistochemical; VB, venous blood; AML, acute myeloid leukemia; BM, bone marrow; ALP, alkaline phosphatase; ANBE, α-naphthyl butyrate esterase; CAE, chloroacetate esterase; MPx, myeloperoxidase*.

*Underlined dog numbers were included in our previous publication on ALP staining in AML (16). Case 2 was also presented in the February 2015 American Society of Veterinary Clinical Pathology on-line rounds (available to members only at https://www.asvcp.org/)*.

*^a^No positive reactions were observed for MPx in those cases in which this cytochemical staining*.

*^b^Additional rationale for classification of AML subtype is provided: (1) for mixed lineage #2, flow cytometric and cytochemical staining results in bone marrow supported a monoblastic lineage, but negative results for cytochemical stains and morphologic features supported an erythroid lineage in spleen; (2) for suspect M5 #4, the cells lacked flow cytometric markers of myeloid differentiation and only expressed strong ALP on cytochemical staining, which is not expressed in normal mature or immature neutrophils in dogs and supports an AML (16). The lack of expression of the neutrophil enzyme CAE in blasts on cytochemical staining argues against a myelomonocytic classification despite the presence of ≥20% mature and immature neutrophils in blood (bone marrow not available for evaluation); (3) for suspect M5 #9, morphologic features and strong positive ALP staining supported monocytic differentiation; (4) for suspect M4 #11, the cells expressed neutrophil or monocyte differentiation antigens on flow cytometry and the percentage of CAE-positive cells on cytochemical staining was close to 20%; (5) for mixed lineage #20, there was concurrent expression of B (CD22, this was not confirmed by IHC for Pax-5 on a core biopsy) and T (CD3, this was not confirmed on IHC staining of a core biopsy with CD3) cell markers with flow cytometry and myeloid markers on cytochemical staining (diffuse light or chunky ANBE, CAE positive); (6) for mixed lineage #23, there was morphologic (micromegakaryocytes), flow cytometric (CD61), and cytochemical evidence (diffuse light or chunky ANBE) of megakaryocytic differentiation, but cells also expressed ALP (lacking in normal megakaryocytes and platelets in the dog) and showed morphologic features of myeloid differentiation. The tumor cells also expressed CD3*.

*^c^#21: limited flow cytometric panel done (only conjugated antibodies); #22: insufficient blasts on the blood smear to evaluate 100 cells on cytochemical staining; #25: flow cytometric analysis on the lymph node was done at CSU at presentation to the oncologist. The dog was euthanized 2.5 months after diagnosis of and treatment for AML and venous blood and bone marrow were submitted to Cornell University for morphologic assessment, flow cytometric analysis, and cytochemical staining. At the time of euthanasia, the dog had a total leukocyte count of 79.8 × 10^6^/L, consisting of 39.1 × 10^6^/L blasts (a few with purple cytoplasmic granules), with 32.7 × 10^6^/L monocytes (some dysplastic) and 0.8 × 10^6^/L neutrophils in blood*.

Bone marrow aspirates were done in 14 dogs, all of whom had a blast count >20%, compatible with a diagnosis of acute leukemia (17). In one of these dogs, the BM aspirate was done after the dog had been treated with AML for 2.5 months (#25). The dog stopped responding to treatment and was euthanized, at which time blood and BM aspirates were sent to Cornell University for morphologic assessment, flow cytometric analysis, and cytochemical staining. These results supplemented the original results obtained from lymph node aspirates (flow cytometry done at CSU; Table 6). Tissue infiltrates (liver, spleen, lymph nodes) or involvement of body cavities (abdomen and thorax) were seen in 10 dogs on aspiration. Dysplasia was evident in cells in the BM, body cavity, or tissue aspirates of five dogs. On flow cytometric labeling, most of the tumor cells in the dogs with AML expressed CD45, CD18, and CD34 with aberrant negative expression of MHCII, as previously reported (16). CD34 expression was negative in two cases, both of which were subtyped as likely acute myelomonocytic leukemia. In 22 dogs in which flow cytometry was performed, tumor cells in 15 dogs lacked expression of T (CD3, CD5) or B (CD21, CD22) cell markers. Tumor cells from four dogs weakly expressed CD5, but the cells in three of these four dogs expressed other myeloid antigens, such as CD11b, and all had strong positive reactions for ALP or other myeloid enzymes (diffuse light or chunky ANBE, CAE) and were negative for CD3 and TCRαβ, supporting a diagnosis of AML over acute lymphoid leukemia (ALL). Similarly, two cases expressed CD3 but not CD5 or TCRαβ; the CD3 expression in one of these cases was not confirmed on IHC staining of a core biopsy of the BM (#20). The latter case of mixed lineage AML was also positive for CD22, but negative for Pax-5 on IHC staining of the marrow core sections. CD22, but not CD21, was expressed on a case of myelomonocytic AML (#16) (Table 6).

Clonal rearrangements in T or B cell receptors were seen in 16 dogs (64%). Of these, six dogs had clonal arrangements in both T and B (mostly with primer set 2) cell receptors (biclonal), six dogs had only a clonally rearranged T cell receptor (DNA quantity was insufficient to identify B cell clonality in one of these dogs) and four dogs had only a clonally rearranged B cell receptor (mostly with primer set 2). In one dog (#6), clonality testing was performed at both Cornell University and CSU as part of internal verification studies and yielded the same result of T cell clonality.

## Discussion

Our results indicate that a high proportion of dogs with AML (64% in this cohort) can express clonally rearranged CDR3 regions in B or T cell receptors. This result indicates that clonality testing should not be relied upon for distinguishing between AML and lymphoid neoplasms, such as ALL or lymphoma with a secondary leukemia, as previously recommended (2). Recently, Keller and colleagues have also advocated against the use of clonality testing for distinguishing B from T cell neoplasms (1). However, in many cases of lymphoid neoplasia, the tumor shows fidelity between clonality and other phenotyping tests (IHC or flow cytometry) (2, 6, 20), and clonality assessment may still be useful, at the very least for confirming neoplasia, in sites where sufficient cells cannot be retrieved for other phenotyping techniques or invasive diagnostic procedures are contraindicated (12).

Our results of clonally rearranged lymphoid receptors are similar to that reported previously in one of three dogs (3) and people with AML. In 4 studies of patients with AML diagnosed with cytochemical staining using traditional French-American-British working group criteria (21), 7% of 14 patients had a clonal T cell rearrangement (22), 36% of 25 patients had B (*n* = 3), T (*n* = 4), or biclonal (*n* = 2) rearrangements (23), 13% of 24 patients had clonal T (*n* = 2) or biclonal (*n* = 1) rearrangements (24), and 40% of 35 patients had clonal B cell rearrangements (T cell clonality not tested) (25). Of note, in three of these studies of human patients with AML, tumor cells aberrantly expressed T or B cell antigens; however, expression of lineage-associated antigens was not associated with clonality of the same lineage in all cases (23–25). We found a similar result in this study, in which tumor cells in dogs with AML expressed B or T cell surface antigens, yet displayed non-clonal, biclonal, or B or T cell receptor clonal rearrangements. Although non-clonal or polyclonal results on clonality testing have been used to support a diagnosis of AML in previous reports (6, 10, 14, 15), false-negative reactions for clonality do occur in lymphoid neoplasms, with reported sensitivities ranging from 67 to 98% (5–7, 10, 20), using similar primer sets to that used herein.

Dogs with AML had a high frequency of biclonal rearrangements, i.e., 38% of those cases with clonal rearrangements (6/16) or 24% of this cohort of 25 dogs. Clonal rearrangements in both B and T cell receptors have been reported in dogs with lymphoid neoplasms; however, the proportion of biclonality in dogs with AML is higher than that reported for dogs with lymphoid neoplasia, which ranges from 1 to 10% (3, 5, 6, 10). These data indicate that if clonality testing is done in a dog with acute leukemia of unclear phenotype, a B and T cell biclonal result should raise the index of suspicion for an underlying AML and prompt further testing for AML, such as expanded testing for myeloid antigen expression on flow cytometric analysis or cytochemical staining. Similarly, a diagnosis of AML should be considered with a non-clonal test result, particularly if the leukemia lacks expression of B or T cell markers with flow cytometry and is positive for the stem cell marker CD34 and negative for MHCII. The latter results are characteristic findings in dogs with AML (16) but unusual with lymphoid neoplasms, which frequently express MHCII and often lack CD34 (4, 16). However, there are known variants of T and B cell lymphoma or leukemia that drop off MHCII expression (26, 27) or that express CD34 (13, 16). Furthermore, two dogs (both likely myelomonocytic leukemia, #5 and #11) lacked CD34 and MHCII expression, as we have previously reported for this subtype of AML (16), indicating that not all AML express CD34.

A limitation of this study is that we used less-sensitive techniques and “older” primer sets for performing clonality assessment at our institution. We unfortunately still do not have the equipment to perform more sensitive assays, and there is insufficient material remaining to submit non-clonal samples to other sites for testing. Thus, it is possible that some of the cases that were classified as non-clonal would have been reclassified as clonal if newer primer sets or more sensitive techniques were used (1, 2, 6, 7, 28). A lower sensitivity of clonality detection in this study could mean that an even higher proportion of dogs with AML had clonal rearrangements. In addition, the assessment of clonality with high-resolution melt curve analysis is subjective, and there are more quantitative techniques that can be done with this method to reduce subjectivity (10). However, we always performed confirmatory polyacrylamide gel electrophoresis with equivocal or inconclusive results for the high-resolution melt curve analysis and defaulted to the results of the gel if there were discrepancies in interpretation. Because a direct comparison between quantitative and subjective assessments was not made in the aforementioned study (10), it remains to be determined if quantitation is truly superior. It is intriguing that clonality in the CDR3 region of the B cell receptor was detected more frequently with primer set 2 than primer set 1. These primer sets differ with the primer that amplifies the junctional region of CDR3 (3), raising the possibility that this area of the B cell receptor shows more frequent clonal rearrangements than the junctional region amplified by primer set 1. Sequencing of relevant regions of the DNA in dogs with AML may prove informative and lead to the design of primers that are more specific for AML versus B cell neoplasms. However, this theory remains to be tested in future studies.

Dogs in this study were typically older, as reported for other studies of AML in dogs (29, 30), but AML can occur in dogs as young as 1–2 years (31). Similar to two other studies, German Shepherd dogs were one of the more common affected breeds (30, 31), which may represent a true breed disposition to AML or breed popularity. It is notable that several dogs presented with lymphadenopathy (peripheral or internal, solitary, or multiple nodes), organomegaly (spleen, liver, or both), or body cavity effusions, signs that are more typical for lymphoma than acute leukemia. Indeed, a presumptive diagnosis of lymphoid neoplasia was made before referral or after cytologic examination of body cavity fluid or tissue aspirates in six dogs (#8, 17, 20, 21, and 25). It was only after flow cytometric analysis or cytochemical staining was performed that the diagnosis was changed to AML. These results indicate that AML can mimic lymphoma in clinical presentation and, although less common than lymphoma, should still be considered as a differential diagnosis for animals presenting with lymphadenopathy or organomegaly, particularly if the organ enlargement is mild to moderate or if there are concurrent cytopenias or blasts identified in blood smears.

Most of the dogs in this study had bicytopenia or pancytopenia and an obvious leukemia with >20% blasts in peripheral blood. However, some dogs had a regenerative anemia, and one dog (#8) had a normal hemogram with no evidence of circulating tumor cells. The latter dog was diagnosed with a non-B non-T lymphoma before referral to our institution and had lower numbers of marrow blasts than is typical for dogs with AML, in which >70% blasts or marrow effacement is usually seen. Thus, a normal hemogram or regenerative anemia does not rule out a diagnosis of AML. In some cases, tumor cells in blood smears were presumptively identified as “lymphocytes” or as “lymphoblastic leukemia,” which may lead to an erroneous diagnosis of lymphoid neoplasia. It is difficult to impossible to determine if blasts are myeloid or lymphoid in origin on the basis of morphologic criteria alone, although features of myeloid differentiation can be apparent in some neoplastic cells and dysplasia in hematopoietic cell lineages can be additional support (but not confirmatory) for an underlying AML. Thus, at our institution, we identify immature neoplastic cells as generic “blasts” and rely on combined results from morphologic assessment, immunophenotyping, and cytochemical assays to distinguish between AML and lymphoproliferative disorders (16).

In contrast to human medicine (17, 18), there are currently no standardized criteria for diagnosis of AML in animals. In the past, the diagnosis of AML was based primarily on morphologic features and cytochemical staining (32). With the increasing availability of cross-reactive or canine-specific reagents for identification of lineage-associated antigens, immunophenotyping with flow cytometric analysis has largely supplanted cytochemical staining for diagnosis of AML (29, 30). A limitation of this approach is the lack of commercial reagents that can identify earlier myeloid-associated antigens, such as CD15, CD33, and CD123, in dogs, with most available antibodies being restricted to stem cell antigens and late stage differentiation antigens, such as CD14. For this reason, we used the presence of >20% blasts in blood or BM, body cavity fluid, or tissue aspirates and a combination of morphologic criteria, flow cytometric, and cytochemical results to confirm a diagnosis of AML in this study, as we have described previously (16). Dogs that did not have >20% blasts in blood had >20% blasts in marrow or aspirates of tissues or body cavity fluids. In this study, the combination of different tests was required for a diagnosis of AML, because the tumor cells in some dogs with AML displayed lineage infidelity or aberrant expression patterns and expressed B or T cell antigens, particularly CD5, on flow cytometric analysis, as we have previously reported (16). Aberrant expression of lymphoid antigens is also seen in human patients with AML (23–25, 33). In addition, positive cytochemical reactions for ALP or ANBE are not specific for AML, because these enzymes can be expressed in lymphoid tumors (16, 34, 35). Similarly, there have been rare reports of focal cytoplasmic reactivity for CAE in lymphocytes (35). For this reason, we used the preponderance of evidence, specifically the lack of or weak expression of lymphoid markers and MHCII with expression of myeloid-associated and stem cell antigens, and cytochemical staining more typical of myeloid cells (strong ALP, diffuse light or chunky ANBE, CAE) to make a diagnosis of AML over ALL or lymphoma. We have found that CD11b and strong ALP expression are particularly useful markers of AML, because lymphoid neoplasms are typically negative for CD11b and only weakly express or do not express ALP (16). However, neither of these markers are 100% specific for AML. Thus, it is still possible that some of cases in this study that lacked expression of myeloid-associated antigens but were diagnosed as AML on the basis of morphologic features and cytochemical staining patterns (#4 and #9) were in fact ALL. In five dogs, there were discrepant results from the flow cytometric analysis (#8) or cytochemical staining (#2, #5, #15, and #25) in samples from different sites. The converse was also true in four dogs, which had the same phenotype with flow cytometry (#5 and #10) or cytochemical staining (#8 and #17). Taken together, the results from the dogs with discrepant results between sites suggest that phenotyping, whether by flow cytometric analysis or cytochemical staining, may be more informative if done on all involved sites and not just one site. However, this is not always feasible and may be cost-prohibitive for some patients. It should be noted that there is a move toward standardization of flow cytometric testing for hematopoietic neoplasia in veterinary medicine (36). An *ad hoc* flow cytometric working group has also been established by clinical pathologists in the American Society of Veterinary Clinical Pathology. This group is working toward defining a minimum recommended flow cytometric panel of conjugated antibodies that can be used across institutions worldwide for diagnosis of hematopoietic neoplasia.

Due to the lack of genetic abnormalities that characterize newer AML classifications (17, 18), we used standard criteria to subtype the AML in this study, which included morphologic assessment and cytochemical staining reactions. These criteria were originally established by the French–American–British working group (21) and are now defined as AML – not otherwise specified by WHO (17, 18). Most of the dogs in this study (14/25, 56%) were subtyped as AML-M5 or monoblastic/monocytic leukemia, which is one of the more common subtypes in some reports of AML in dogs (31, 32). In contrast, another study of AML in dogs have found that myeloid leukemia with minimal differentiation (M0) is the most common subtype (30). However, the latter study used a limited set of markers to classify AML with flow cytometry and did not perform cytochemical staining. In some cases in this study, the classification of AML subtype was based primarily on strong positive cytochemical reactions for ALP (#2, #4, and #9), which we have found to be a useful marker of this subtype of AML. However, it is possible that ALP is a marker of less differentiated earlier myeloid precursors, and some of these cases could be AML with minimal maturation (M0), AML without maturation (M1), or AML with maturation (M2).

In conclusion, the results of our study indicate that clonal rearrangements in B and T cell receptors are a frequent finding in dogs diagnosed with AML. Thus, clonality testing should not be used a tool to discriminate between AML and lymphoid neoplasia (ALL or lymphoma). The current lack of a standardized scheme for diagnosis of AML in dogs creates substantial variability between published studies, making it difficult to compare across them. Establishing new consensus criteria for diagnosis of AML, by updating and incorporating new diagnostic testing modalities into the 1991 classification scheme (32), would be a worthwhile endeavor.

## Author Contributions

TS conceived and designed the study, reviewed and interpreted the data, and wrote the manuscript. GN performed the clonality testing at Cornell University. MS performed and interpreted the cytochemical staining reactions. NB performed and interpreted the flow cytometric analysis. All authors critically reviewed and edited the manuscript.

## Conflict of Interest Statement

The authors declare that the research was conducted in the absence of any commercial or financial relationships that could be construed as a potential conflict of interest.
