# Corrigendum: Dogs with Acute Myeloid Leukemia Have Clonal Re-arrangements in T and B Cell Receptors

**DOI:** 10.3389/fvets.2017.00195

**Published:** 2017-11-14

**Authors:** Tracy Stokol, Gabrielle Nickerson, Martha Shuman, Nicole Belcher

**Affiliations:** ^1^Department of Population Medicine and Diagnostic Sciences, College of Veterinary Medicine, Cornell University, Ithaca, NY, United States; ^2^Animal Health Diagnostic Center, College of Veterinary Medicine, Cornell University, Ithaca, NY, United States

**Keywords:** acute myelogenous leukemia, canine, PARR, clonality testing, phenotyping, leukemia, flow cytometry, cytochemistry

In the original article, there was a mistake in Figure 1 as published. There was a single asterisk placed after the header “Morphologic features of myeloid differentiation in venous blood or bone marrow, body cavity fluid or tissue aspirates” and a double asterisk placed after the header “Myeloid differentiation on flow cytometric analysis.” Both of these symbols referred to more detailed diagnostic criteria provided in Table 1 and are obsolete, since the reader is referred to Table 1 in the legend of Figure 1. The corrected Figure 1, without these symbols, appears below. The authors apologize for this error and state that this does not change the scientific conclusions of the article in any way.

**Figure 1:**
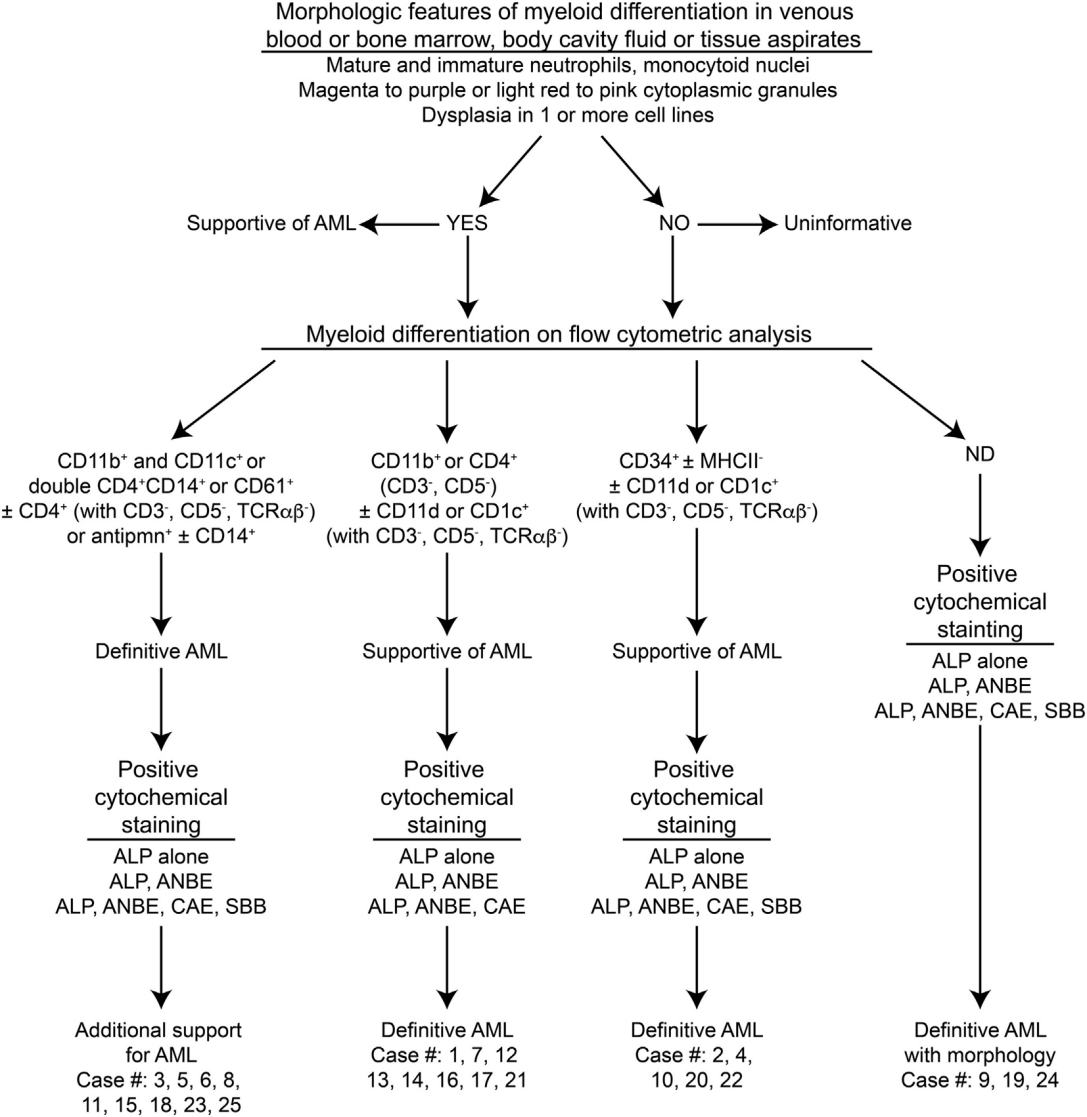
Algorithm used to diagnose acute myeloid leukemia (AML) in the 25 dogs of this study. This diagnostic algorithm was based on the order in which tests were generally performed in our laboratory, i.e., morphologic assessment of blood, bone marrow, or body cavity fluid or tissue aspirates, followed by flow cytometric analysis (performed routinely twice a week), followed by cytochemical staining (performed as needed). After completion of all the tests, the results were reevaluated, and a diagnosis of AML was based on the combined data. The path used to diagnose each case (#) is also shown. More details on the criteria are provided in Table 1.

## Conflict of Interest Statement

The authors declare that the research was conducted in the absence of any commercial or financial relationships that could be construed as a potential conflict of interest.

