# Corrigendum: Dogs with acute myeloid leukemia have clonal rearrangements in T and B cell receptors

**DOI:** 10.3389/fvets.2024.1533729

**Published:** 2025-01-10

**Authors:** Tracy Stokol, Gabrielle A. Nickerson, Martha Shuman, Nicole Belcher

**Affiliations:** ^1^Department of Population Medicine and Diagnostic Sciences, College of Veterinary Medicine, Cornell University, Ithaca, NY, United States; ^2^Animal Health Diagnostic Center, College of Veterinary Medicine, Cornell University, Ithaca, NY, United States

**Keywords:** acute myelogenous leukemia, canine, polymerase testing for antigen receptor rearrangements, clonality testing, phenotyping, leukemia, flow cytometry, cytochemistry

In the published article, there was an error in Figure 1, Table 1, Table 2, Table 3, and Table 6 as published. CD1c in the Figures and Tables should be CD1a. The authors discovered this error after publication of the article when the source of the antibody sent a notification stating that the antigen was not CD1c as originally indicated. The corrected Figure and Tables and their captions appear below.

**Figure 1:**
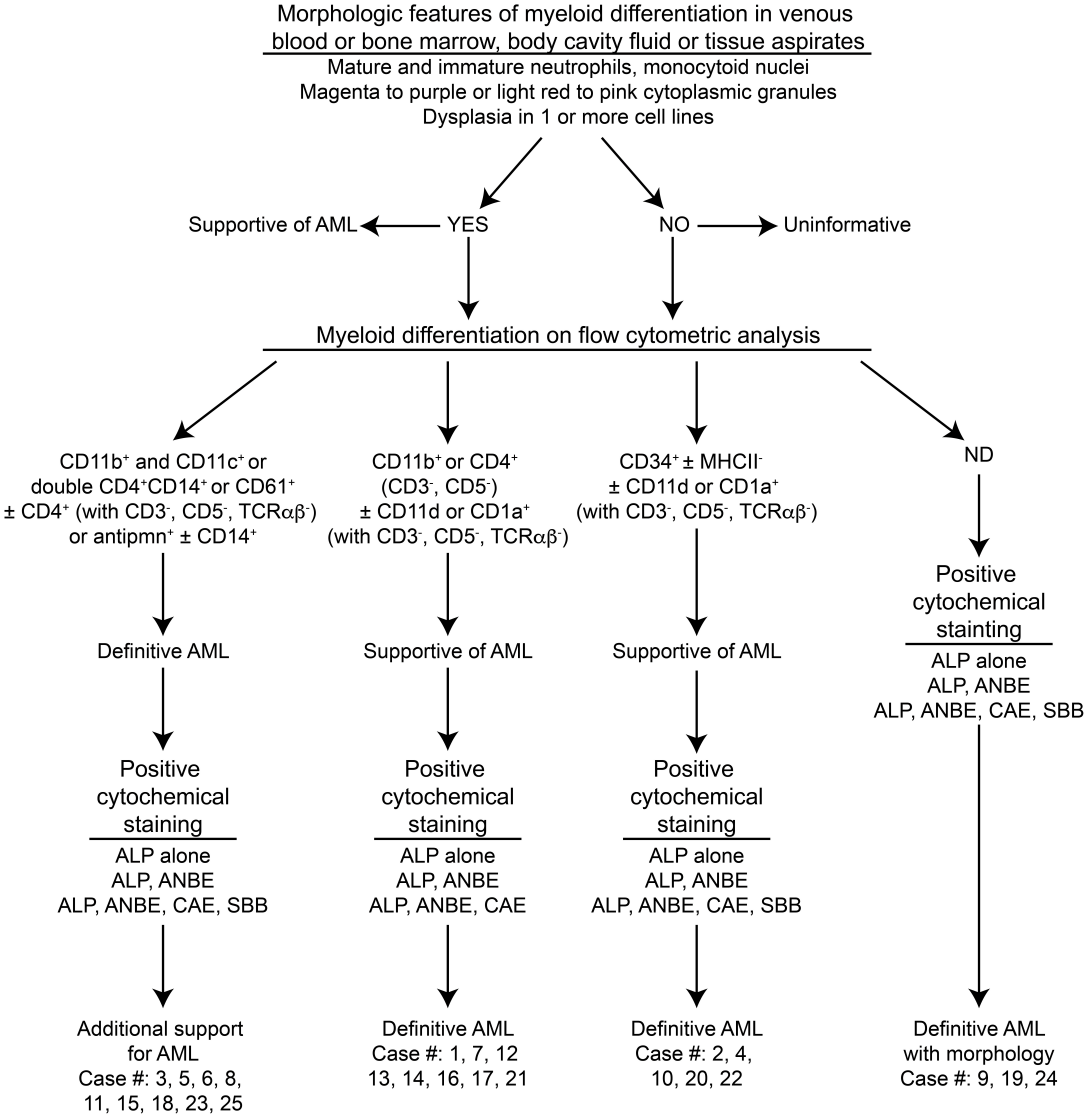
Algorithm used to diagnose acute myeloid leukemia (AML) in the 25 dogs of this study. This diagnostic algorithm was based on the order in which tests were generally performed in our laboratory, i.e., morphologic assessment of blood, bone marrow, or body cavity fluid or tissue aspirates, followed by flow cytometric analysis (performed routinely twice a week), followed by cytochemical staining (performed as needed). After completion of all the tests, the results were reevaluated, and a diagnosis of AML was based on the combined data. The path used to diagnose each case (#) is also shown. More details on the criteria are provided in Table 1.

**Table 1:** Criteria used to support myeloid lineage of leukemia in 25 dogs.

**Test**	**Criteria**
Morphologic features of myeloid differentiation (16, 17)	Neutrophil differentiation (immature and mature neutrophils), monocytoid nuclei, magenta to purple cytoplasmic granules that frequently overlay the nuclei, light red to pink cytoplasmic granules within a light blue cytoplasm, or dysplasia in one or more hematopoietic cell lineages (e.g., giant band neutrophils, neutrophil hypersegmentation, bizarre monocytes, megaloblastic erythroblasts, fragmented or multiple Howell-jolly bodies, giant or abnormally granulated platelets, micromegakaryocytes)
Flow cytometric markers of myeloid differentiation (16)	Neutrophilic differentiation: antineutrophil antibody, monocytic differentiation: CD14, CD11d, or CD1a (the latter two with negative T cell markers), neutrophilic or monocytic differentiation: CD11b, CD11c, or CD4 (the latter with negative T cell markers), megakaryocytes: CD61
Cytochemical stains characteristic of myeloid differentiation (16, 35)	Neutrophils: CAE, MPx, SBB, monocytes: light to strong ALP (monoblasts, differentiating monocytes), diffuse light to chunky ANBE (differentiating monocytes, monoblasts), may be positive for MPx (weaker than neutrophils) or SBB (weaker than neutrophils)

The tests used to define criteria were run sequentially (Figure 1), but the combined results were evaluated after completion of analysis to obtain a definitive diagnosis of AML.

ALP, alkaline phosphatase; AML, acute myeloid leukemia; ANBE, α-naphthyl butyrate esterase; CAE, chloroacetate esterase; MPx, myeloperoxidase; SBB, Sudan Black B.

**Table 2:** Criteria for classification of the subtype of acute myeloid leukemia (AML) based on the World Health Organization scheme – not otherwise specified (17, 18).

**Type of AML**	**Criteria**
Acute myelomonocytic leukemia (M4)	≥20% cells showing neutrophilic differentiation and ≥ 20% showing monocytic (including promonocytes) differentiation. Neutrophil differentiation was based on one or more of the following: • Morphologic features, i.e., mature an immature neutrophils comprised ≥20% cells in blood or bone marrow • Flow cytometry: expression of neutrophil-associated markers such as antineutrophil antibody • Cytochemical staining: Positive for chloroacetate esterase, myeloperoxidase, or Sudan Black B in ≥20% blasts
	Monocytic differentiation was based on one or more of the following: • Morphologic features, i.e., mature and immature monocytes comprised ≥20% cells in blood or bone marrow • Flow cytometry: expression of monocyte-associated markers such as CD14 alone, CD4 and CD14 double positive, or CD11c or CD1a (with negative reactions for T cell markers with the latter) • Cytochemical staining: Positive for alkaline phosphatase (light to strong) or diffuse to light chunky α-naphthyl butyrate esterase
Acute monoblastic or monocytic leukemia (M5)^a^	>80% monocytic lineage (monoblasts, promonocytes, monocytes) based on the above features
Mixed lineage or phenotype	Combination of morphologic features and expression of markers of more than one myeloid lineage or concurrent expression of myeloid and lymphoid lineages on flow cytometry and cytochemical staining with no clear dominant pattern

In some dogs, the final classification was presumptive (suspect) because bone marrow was not available for the classification assays (flow cytometric analysis and cytochemical staining) or one of the classification assays was not done.

^a^Because both neutrophils and monocytes can express CD4 (with negative T cell markers), CD11b, or CD11c, these markers support a diagnosis of AML but alone do not differentiate between subtypes (other criteria were used instead) and are not listed here.

**Table 3:** Antibodies used in flow cytometry at Cornell University to label antigens on tumor cells in liquid samples (blood or bone marrow, body cavity fluid, or tissue aspirates) from dogs with acute myeloid leukemia.

**Antigen**	**Labeled cells**	**Clone**	**Conjugate**	**Source^a^**
CD45	Pan-leukocyte	YKIX716.13	PE	AbD Serotec
CD18	Pan-leukocyte	CA1.4E9	AF647	AbD Serotec
CD3	T cells	CA17.2A12	FITC	AbD Serotec
CD5	T cells	YKIX322.2	PE	AbD Serotec
CD4	T helper/regulatory cells, neutrophils, activated monocytes	YKIX302.9	FITC	AbD Serotec
CD8α	Cytotoxic T cell	YCATE55.9	PE	AbD Serotec
CD28	T cells	B58	APC	eBioscience
TCRαβ	T cells	CA15.8G7	None	UC-Davis
CD21	B cells	B-ly4	PE	BD Biosciences
CD22	B cells	RFB4	PE	Abcam
CD94	Natural killer cell, cytotoxic T cell	HP-3D9	APC	eBioscience
CD14	Monocytes	Tük4	PE	Dako
CD34	Stem cell	1H6	PE	BD Biosciences
MHCII	Lymphocytes, monocytes	YKIX334.2	FITC	AbD Serotec
CD80	Monocytes, neutrophils	16-10A1	APC	eBioscience
CD11b	Neutrophils, monocytes	CA16.3E10	None	AbD Serotec
CD11c	Monocytes, neutrophils,	CA11.6A1	None	AbD Serotec
CD11d	T subset, some monocytes	CA11.8H2	None	AbD Serotec
CD1a	T subset, B subset, monocytes	CA13.9H11	None	UC-Davis
Anti-pmn	Neutrophil	CAD048A	None	VMRD
CD90 (Thy-1)	Lymphocytes, monocytes, stem cells, eosinophils	CA1.4G8	None	UC-Davis
CD61	Platelets	SZ21	PE	Beckman-Coulter

AF, alexafluor; PC, allophycocyanin; FITC, fluorescein isothiocyanate; PE, phycoerythrin; pmn, neutrophil.

^a^Abcam, Cambridge, MA, USA; AbD Serotec, now part of Bio-Rad, Hercules, CA, USA; BD-Biosciences, Franklin Lakes, NJ, USA; Beckman-Coulter, Fullerton, CA, USA; Dako, now part of Agilent Technologies, Santa Clara, CA, USA; UC-Davis: Peter Moore, University of California-Davis, Leukocyte Antigen Biology Laboratory, Davis, CA, USA; VMRD, Pullman, WA, USA.

**Table 6:** Morphologic findings from blood or cytologic smears and results from flow cytometric labeling, cytochemical staining, and clonality testing in 25 dogs with AML.

	**Criteria**					**AML sub-type^b^**	**Clonality**
	**Defining features on venous blood (VB), bone marrow (BM), cavity fluid or tissues**		**Flow cytometric results**		**Cytochemical reactions** ^a^		
**Dog**	**VB**	**BM, body cavity fluid or tissues**	**Positive**	**Negative**			
1	None	ND	VB: CD45, CD18, CD34 (66%), CD11b, CD11d, CD1a, CD90	VB: MHCII, CD3, CD5, TCRαβ, CD21, CD22	VB: ALP (18% light), ANBE (18%), CAE (4%)	M5	VB: B (set 2) and T clonal (Figure 2)
2	ND (only report provided)	BM: 40–45% myeloid blasts, trilineage dysplasia Spleen: >80% blasts (suspect erythroid)	BM: CD45, CD34 (55%), CD11d, CD1a, CD90	BM: MHCII, CD18, CD3, CD5, TCRαβ, CD21, CD22	BM: ALP (64% strong) Spleen: Negative	Mixed lineage	BM slide: T clonal
3	Monocytoid nuclei, purple granules	ND	VB: CD45, CD34 (54%), CD14, CD11b, CD11c, CD11d, CD1a, CD90	VB: MHCII, CD3, CD5, TCRαβ, CD21, CD22	VB: ALP (light), ANBE (8%)	M5	VB slide: T clonal
4	None	ND	VB: CD45, CD34 (82%), CD5 (25%), CD90	VB: CD3, TCRαβ, CD21	VB: ALP (strong)	SuspectM5	VB slide: Non-clonal
5	Trilineage dysplasia	ND	VB: CD45, CD34 (17%), CD11b, CD11c	VB: CD3, CD5, TCRαβ, CD21	ND	M5	VB slide: B clonal (set 2)
6	Monocytoid nuclei, light red granules, dysplasia (mono)	ND LN: >80% blasts.	VB: CD45, CD34 (79%), CD4, CD5 (28%), CD14, CD11b, CD11c, CD11d, CD90	VB: MHCII, CD3, TCRαβ, CD21, CD22	VB: ALP (strong), CAE (7%)	M5	LN slide: T clonal (also CSU)
7	Variable blasts (some monocytoid, others erythroid)	BM: 98% blasts, some with purple granules	BM: CD45, CD34 (79%), CD5 (26%), CD11b, CD1a, CD90	BM: CD3, TCRαβ, CD21	BM: ALP (strong)	M5	BM slide: B (set 2) and T clonal
8	None	BM: 22–30% blasts LN: >90% blasts	BM: CD45, CD18, CD34 (13%), CD11b, CD11c, CD90 LN: CD45, CD18, CD34 (64%), CD90	BM: MHCII, CD3, TCRαβ, CD21, CD22 LN: MHCII, CD3, TCRαβ, CD21, CD22	BM: ALP (>90% strong), ANBE (>90%) LN: ALP (49% moderate), ANBE (39%)	M5	LN slide: Non-clonal
9	Monocytoid nuclei	BM: 57% blasts, monocytoid nuclei	ND	ND	BM: ALP (strong)	Suspect M5	BM slide: Non-clonal
10	Monocytoid nuclei, dysplasia (pmn, mono)	BM: 90% blasts, dysplasia (erythroid, pmn)	VB and BM: CD45, CD34 (29%)	VB and BM: MHCII, CD3, CD5, CD21, CD22	BM: ALP (strong), ANBE, CAE (58%), SBB (6%)	M4	BM slide: B clonal (set 2)
11	None	BM: 35% blasts	BM: CD45, CD18, CD11b, CD11c	BM: MHCII, CD34 (5%), CD3, CD5, TCRαβ, CD21, CD22	BM: ALP (60% strong), ANBE (18%), CAE (18%)	Suspect M4	BM slide: T clonal, B inconclusive
12	Trilineage dysplasia	ND	VB: CD45, CD18, CD34 (98%), CD4, CD11b, CD90	VB: MHCII, CD3, CD5, TCRαβ, CD21, CD22	VB: ALP (100% strong), ANBE (100%)	M4	VB slide: B clonal (set 2)
13	None	BM: 99% blasts LN: >90% blasts	BM: CD45, CD18, CD34 (97%), CD11b, CD11d, CD1a, CD90	BM: MHCII, CD3, CD5, TCRαβ, CD21, CD22	BM: ALP (100% strong), ANBE (4%)	M5	BM slide: B (set 1) and T clonal
14	None	BM: 83% blasts with pink granules	BM: CD45, CD18, CD34 (18%), CD11b	BM: MHCII, CD3, CD5, TCRαβ, CD21, CD22	BM: ALP (3% moderate), ANBE (29%), CAE (12%)	M5	BM slide: Non-clonal
15	Red to purple granules	BM: >90% blasts	VB: CD45, CD34 (36%), CD18, CD14, CD11b, CD11c, CD1a, CD90	VB: MHCII, CD3, CD5, TCRαβ, CD21, CD22	VB: ALP (54% moderate), ANBE (24%) BM: ALP (100% strong), ANBE (34%), CAE (12%)	M5	VB slide: B (set 2) and T clonal
16	Magenta to purple granules	BM: >90% blasts	BM: CD45, CD18, CD34 (95%), CD22 (71%), CD11b, CD11d, CD90	BM: MHCII, CD3, CD5, TCRαβ, CD21	BM: ALP (100% strong), ANBE (99%), CAE (21%)	M4	VB slide: T clonal
17	None	BM: ND PLF: >80% blasts	VB: CD45, CD34 (37%), CD5 (34%), CD11b, CD90	VB: MHCII, CD3, TCRαβ, CD21, CD22	VB: ALP (100% moderate), ANBE (54%), CAE (4%) PTF: ALP (100% moderate), ANBE (7%), CAE (7%)	M5	VB and PTF slides: Non-clonal
18	None	ND	VB: CD45, CD18, CD34 (91%), CD4, CD11b, CD11c, CD11d, CD90	VB: MCHII, CD3, CD5, TCRαβ, CD21, CD22	VB: ALP (11% moderate)	M5	VB slide: B (set 2) and T clonal
19	None	BM: 99% blasts	ND	ND	VB: ALP (100% strong), ANBE (33%), CAE (4%)	M5	BM slide: Non-clonal
20	None	BM: 32-70% blasts	BM: CD45, CD18, CD34 (94%), CD3 (35%), CD22 (35%)	BM: MHCII, CD5, TCRαβ, CD21	BM: ANBE (>80%), CAE (59%)	Mixed lineage	BM slide: B clonal (set 2)
21^c^	None	BM: ND LN: >80% blasts, magenta granules	LN: CD45, CD34 (36%), CD4	LN: MHCII, CD3, CD5, CD21, CD22	LN: ALP (75% strong), ANBE (4%)	M5	LN slide: B clonal (set 1)
22^c^	Dysplasia (pmn, eos, platelets)	BM: ND LN: 27% blasts, dysplasia (pmn, eos)	VB: CD45, CD18, CD34 (39%) and MHCII double positive, CD1a	VB: CD3, CD5, TCRαβ, CD21, CD22	VB: ALP (>80%), ANBE (33%), CAE (33%)	M4	LN slide: Non-clonal
23	Trilineage dysplasia	BM: ND PTF: >20% blasts	VB: CD45, CD18, CD34 (6%), CD3 (62%), CD61 (60%)	VB: MHCII, CD5, TCRαβ, CD21, CD22	VB: ALP (100% moderate), ANBE (9%)	Mixed lineage	PTF fluid: T clonal
24	None	ND	ND	ND	VB: ALP (98% moderate), ANBE (24%), CAE (24%)	M4	VB slide: Non-clonal
25	Monocytoid nuclei, dysplasia (mono)	BM: 95% blasts LN: >20% blasts, monocytoid nuclei, dysplasia (mono)	VB: CD45, CD18, CD34 (41%), CD4, CD14 (26% double positive with CD4), CD11c, CD11d, CD1a, CD90 BM: ND LN: CD34 (6%), CD14 and CD4 double (CSU)	VB: MHCII, CD3, CD5, TCRαβ, CD21, CD33 BM: ND LN: MHCII, CD3, CD5, CD21 (CSU)	VB: ALP (63% light), ANBE (90%), CAE (35%) BM: ALP (85% moderate), ANBE (68%), CAE (44%) LN: ALP (65% moderate), CAE (17%)	M4	LN slide: B and T clonal (CSU)

All results were obtained from testing done at Cornell University unless stated otherwise in parentheses. Note that not all of the antibodies provided in Table 3 were used in every dog, and negative results for flow cytometric analysis was confined to T (CD3, CD5, TCRαβ), B (CD21, CD22), or stem (CD34) cell markers, the pan-leukocyte marker (CD18), and MHCII. If results for the latter markers are not provided, then the antibody against the marker was not tested in that case. If a marker or cytochemical reaction is not listed, it means the test result was negative (usual scenario) or not performed. When available, the percentage of blasts with positive cytochemical reactions (and the strength of the reaction for ALP) is provided. NA, not available for review with no report provided; ND, not done; PLF, pleural fluid; PTF, peritoneal fluid; M4, acute myeloid leukemia – not otherwise specified, myelomonocytic; M5, acute myeloid leukemia – not otherwise specified, monocytic or monoblastic; CSU, Colorado State University; pmn, neutrophil; mono, monocyte; eos, eosinophil; IHC, immunohistochemical; VB, venous blood; AML, acute myeloid leukemia; BM, bone marrow; ALP, alkaline phosphatase; ANBE, α-naphthyl butyrate esterase; CAE, chloroacetate esterase; MPx, myeloperoxidase. Underlined dog numbers were included in our previous publication on ALP staining in AML (16). Case 2 was also presented in the February 2015 American Society of Veterinary Clinical Pathology on-line rounds (available to members only at https://www.asvcp.org/).

^a^No positive reactions were observed for MPx in those cases in which this cytochemical staining.

^b^Additional rationale for classification of AML subtype is provided: (1) for mixed lineage #2, flow cytometric and cytochemical staining results in bone marrow supported a monoblastic lineage, but negative results for cytochemical stains and morphologic features supported an erythroid lineage in spleen; (2) for suspect M5 #4, the cells lacked flow cytometric markers of myeloid differentiation and only expressed strong ALP on cytochemical staining, which is not expressed in normal mature or immature neutrophils in dogs and supports an AML (16). The lack of expression of the neutrophil enzyme CAE in blasts on cytochemical staining argues against a myelomonocytic classification despite the presence of ≥20% mature and immature neutrophils in blood (bone marrow not available for evaluation); (3) for suspect M5 #9, morphologic features and strong positive ALP staining supported monocytic differentiation; (4) for suspect M4 #11, the cells expressed neutrophil or monocyte differentiation antigens on flow cytometry and the percentage of CAE- positive cells on cytochemical staining was close to 20%; (5) for mixed lineage #20, there was concurrent expression of B (CD22, this was not confirmed by IHC for Pax-5 on a core biopsy) and T (CD3, this was not confirmed on IHC staining of a core biopsy with CD3) cell markers with flow cytometry and myeloid markers on cytochemical staining (diffuse light or chunky ANBE, CAE positive); (6) for mixed lineage #23, there was morphologic (micromegakaryocytes), flow cytometric (CD61), and cytochemical evidence (diffuse light or chunky ANBE) of megakaryocytic differentiation, but cells also expressed ALP (lacking in normal megakaryocytes and platelets in the dog) and showed morphologic features of myeloid differentiation. The tumor cells also expressed CD3.

^c^#21: limited flow cytometric panel done (only conjugated antibodies); #22: insufficient blasts on the blood smear to evaluate 100 cells on cytochemical staining; #25: flow cytometric analysis on the lymph node was done at CSU at presentation to the oncologist. The dog was euthanized 2.5 months after diagnosis of and treatment for AML and venous blood and bone marrow were submitted to Cornell University for morphologic assessment, flow cytometric analysis, and cytochemical staining. At the time of euthanasia, the dog had a total leukocyte count of 79.8 × 10^6^/L, consisting of 39.1 × 10^6^/L blasts (a few with purple cytoplasmic granules), with 32.7 × 10^6^/L monocytes (some dysplastic) and 0.8 × 10^6^/L neutrophils in blood.

The authors apologize for this error and state that this does not change the scientific conclusions of the article in any way. The original article has been updated.

